# Win Ratios in Ophthalmology—a Hierarchical Metric to Evaluate Adverse Outcomes in Comparative Studies

**DOI:** 10.1016/j.xops.2026.101382

**Published:** 2026-08-25

**Authors:** Jeremy C.K. Tan, George Kong, David Wechsler, David Manning, Hamish Dunn, Brian Chua, Helen Danesh-Meyer, Louis Arnould, Gus Gazzard, Mitchell Lawlor

**Affiliations:** 1 Save Sight Institute, Faculty of Medicine and Health, University of Sydney, Sydney, New South Wales, Australia; 2 School of Clinical Medicine, University of New South Wales, Kensington, New South Wales, Australia; 3 Department of Ophthalmology, Prince of Wales Hospital, Randwick, New South Wales, Australia; 4 Glaucoma Investigation and Research Unit, Royal Victorian Eye and Ear Hospital, East Melbourne, Victoria, Australia; 5 Centre for Eye Research Australia, The University of Melbourne, Parkville, Victoria, Australia; 6 Department of Glaucoma, Sydney Eye Hospital, Sydney, New South Wales, Australia; 7 Faculty of Medical and Health Sciences, University of Auckland, Auckland, New Zealand; 8 Department of Ophthalmology, Dijon University Hospital, Dijon, France; 9 Pathophysiology and Epidemiology of Cerebro-Cardiovascular Diseases (PEC2), (EA 7460), Faculty of Health Sciences, Université de Bourgogne, Dijon, France; 10 National Institute for Health and Care Research Biomedical Research Centre at Moorfields Eye Hospital National Health Service Foundation Trust and University College London Institute of Ophthalmology, London, United Kingdom

**Keywords:** Minimally invasive glaucoma surgery, Glaucoma filtration surgery, Xen45 gel stent, Trabeculectomy

## Abstract

**Purpose:**

Clinical trials in ophthalmology frequently report multiple efficacy and safety outcomes of varying clinical importance analyzed either in isolation or as simple composites. The Win Ratio (WR) is a hierarchical composite metric originally developed to address such limitations in cardiovascular research by integrating outcomes according to their clinical priority. In this study, we assess its potential utility in ophthalmology outcomes with glaucoma surgery as the exemplar.

**Design:**

Retrospective registry-based study.

**Subjects:**

There are 1257 eyes that underwent a Xen gel stent (Xen) implantation or trabeculectomy (Trab) with a minimum 12 months of follow-up.

**Methods:**

Propensity score matching yielded two comparable cohorts (212 eyes each) without significant differences in age, baseline intraocular pressure, number of medications, visual acuity, or visual field mean deviation. A predefined hierarchy of eight adverse outcomes was constructed, headed by loss of light perception vision, secondary glaucoma surgery, followed by early postoperative complications (e.g., hypotony and hyphaema) or bleb needling. Each Xen-Trab pair was evaluated sequentially and classified as a win, loss, or tie.

**Main outcome measures:**

The overall WR with 95% confidence intervals (CIs) and *P* value was estimated, and sensitivity analyses examined the robustness of results under alternative outcome hierarchies and random permutations.

**Results:**

The Xen group accumulated fewer wins than Trab (WR 0.83, 95% CI 0.63–1.10, *P* = 0.19), although the difference was not significant. Differences were driven by higher rates of secondary glaucoma surgery (18.4% vs. 12.7%) and bleb needling within the first 6 months (28.8% vs. 13.2%) in the Xen group, with remaining outcomes occurring infrequently and evenly distributed in each group. Sensitivity analyses with alternative hierarchies yielded consistent results (WR range 0.80–0.98), and random permutations of lower-ranked outcomes clustered around WR 0.77, supporting the consistency of the final outcome.

**Conclusions:**

The WR offers a clinically meaningful, hierarchical approach to comparing multidimensional treatments that respects clinical priorities. Our findings illustrate the potential utility of this metric in the prospective evaluation of trial outcomes in ophthalmology.

**Financial Disclosure(s):**

The authors have no proprietary or commercial interest in any materials discussed in this article.

Interventional trials in ophthalmology frequently report a range of efficacy and safety outcomes of varying clinical importance, analyzed either separately or as simple composites that place equal emphasis on all components.^1^, ^2^, ^3^ In glaucoma filtration surgery (GFS), for example, efficacy measures may include intraocular pressure (IOP) or medication reduction, and safety events include loss of light perception vision, the need for secondary surgical interventions, and postoperative complications such as endophthalmitis. Surgical failure is often defined as inadequate IOP reduction or the occurrence of an aforementioned safety event and is commonly analyzed using time-to-first-event survival analyses. This approach may not, however, adequately reflect the hierarchy of clinical priorities or differing severity of end points; for instance, loss of light perception is an arguably more severe outcome than requiring secondary surgery or numerical hypotony. In addition, attempts to construct simple composite outcomes can produce misleading impressions of treatment benefit. For instance, in the Zhongshan Angle Closure Prevention clinical trial for angle-closure disease,^4^ the primary outcome was a composite of three study end points—IOP above 24 mmHg on two separate occasions, at least 1 clock of peripheral anterior synechiae (PAS), or an episode of acute angle closure. While the between-group difference was driven almost entirely by PAS formation, PAS formation is of lesser clinical relevance than the other two end points. Removing PAS from the composite outcome eliminated the statistical significance of the difference between groups, highlighting how composites can mask the true clinical significance of their components.^5^

The Win Ratio (WR), first proposed by Pocock et al in 2012,^6^ was developed to address the limitations of composite outcomes, and has since been widely adopted in cardiovascular research. In this method, each patient in 1 treatment arm is paired with one or more patients in the reference arm, and each pair is compared hierarchically. The comparison in glaucoma begins with the most clinically significant outcome, such as loss of light perception, and only moves to the next outcome in the hierarchy if the pair is tied on the previous one. Within each pair, the patient with the better outcome is counted as a “win” and the other as a “loss”; if both remain identical across all outcomes, the pair is classified as a tie. After evaluating all cross-arm pairs, the total number of wins and losses is tallied, and their ratio forms the WR, representing the overall advantage of 1 treatment over the other. This framework prioritizes serious events over less critical ones, can integrate both efficacy and safety end points, and yields an intuitive measure of overall treatment effect expressed as the ratio of wins to losses. Its uptake in cardiovascular trials has demonstrated the method’s clear value in producing more clinically meaningful interpretations of complex datasets with multiple outcomes of differing priorities.^7^ Given the parallel challenges in ophthalmology, where treatments often involve competing risks and outcomes of unequal clinical importance, the WR may provide a similarly valuable analytic approach.

In this study, we applied the WR methodology to a large registry-based cohort of patients who underwent either Xen gel stent implant (Xen) or trabeculectomy (Trab) surgery. Our objective was to investigate the potential utility of the WR as a metric in the prospective evaluation of trial outcomes in ophthalmology by comparing the safety profile of this minimally invasive bleb surgery device to the standard of care in GFS.

## Methods

### Study Design and Population

We conducted a retrospective analysis using data from the Fight Glaucoma Blindness Registry, an international, web-based clinical registry designed to collect high-quality clinical data on glaucoma management. The registry captures mandatory baseline fields to accurately phenotype patients, and then mandatory fields at each follow-up visit, including best-corrected visual acuity (BCVA), IOP, treatments administered, adverse events, and any additional procedures performed as previously described.^8^, ^9^, ^10^ Ethics approval was provided by the Royal Australian and New Zealand College of Ophthalmology Human Research Ethics Committee, with waiver of consent due to the retrospective nature of the study. The study adhered to the Declaration of Helsinki. Consecutive adult patients (≥18 years of age) who underwent either Xen or Trab with intraoperative mitomycin-C without concurrent cataract surgery, and who had at least 12 months of postoperative follow-up data, were included in this analysis.

#### Baseline Variables and Propensity Score Matching

Baseline variables included age, gender, diagnosis subtype, central corneal thickness, visual-field mean deviation (VF MD), and BCVA. To ensure fairness in baseline characteristics between comparator arms, eyes undergoing Xen and Trab were matched 1:1 using propensity scores based on age, baseline IOP, number of medications, BCVA, and VF MD. Matching was performed using a nearest-neighbor algorithm without replacement,^11^ yielding two balanced cohorts of 212 eyes each. Matching was also performed only on subjects with complete baseline data. Baseline comparability was assessed with standardized mean differences and two-sample statistical tests.

#### Primary outcome—pairwise Hierarchical Comparison of Adverse Events

The primary outcome was the pairwise hierarchical comparison of adverse events between Xen and Trab, analyzed using the WR method (Figure 1). Outcomes were initially ranked by clinical importance, with vision-threatening and irreversible events considered first. This ordering ensures that vision-threatening events dominate the analysis, while less severe complications are only considered if more severe complications are similar. Although it is feasible to include one or more of the efficacy success criteria within the hierarchical structure, for the purpose of this proof of concept study, we focused only on complications and adverse events.

**Figure 1:**
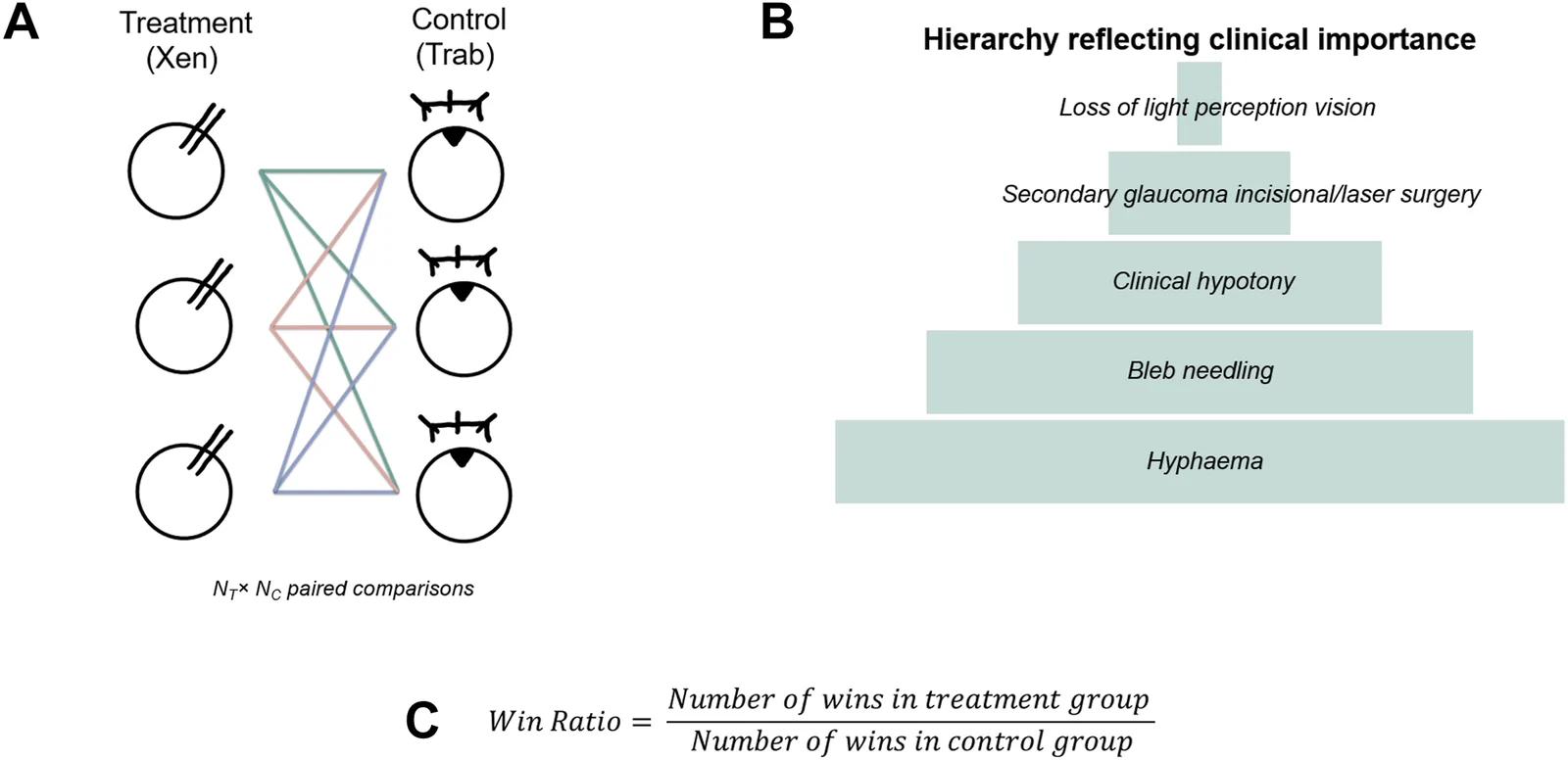
The Win Ratio is a statistical measure used to compare outcomes between two groups—often treatment vs. control—in clinical trials, especially when evaluating composite endpoints. **A**: The method involves pairwise comparisons between all eyes in the treatment (e.g., Xen) and control (e.g., Trab) groups. **B**: For each pair, outcomes are assessed in a predefined order of clinical importance from top to bottom. The group whose eye has a better outcome at the first level of difference is awarded a “win.” If the two eyes are tied at the first level (e.g., both have or both avoid the event), the comparison proceeds to the next level. This continues through the hierarchy until a win/loss is determined or the pair is declared a tie if outcomes are identical or noninformative at all levels. **C**: The overall ratio is calculated as the total number of wins in the treatment group divided by the total number of wins in the control group. Trab = trabeculectomy; Xen = Xen gel stent implant.

The prespecified hierarchy of outcomes chosen for this analysis was:

1. Loss of light perception vision
2. Secondary glaucoma surgery
3. Hypotony (IOP <6) with VA loss ≥10 letters (0-6 months; within the first 6 postoperative months)
4. Hypotony maculopathy (0–6 months)
5. Choroidal effusions (0–6 months)
6. Numerical hypotony (IOP <6 without ≥10 letters VA loss) (0–6 months)
7. Hyphaema with VA loss ≥10 letters (0–6 months)
8. Bleb needling (0–6 months)

#### Concept of WR

The WR is based on pairwise comparisons. Each Xen eye was compared with each Trab eye, yielding 44 944 possible comparisons. For each pair, the most clinically important outcome was assessed first (e.g., loss of light perception). For time-to-event outcomes (loss of light perception, secondary glaucoma surgery), the eye with the longer event-free survival time was designated the “winner.” For binary outcomes (postoperative complications within the first 6 months), the eye without the complication was designated the “winner.” If both eyes were tied for an outcome (e.g., both remained event-free for the same duration, or both experienced the same binary event), the analysis proceeded to the next outcome in the hierarchy until a winner was identified or all outcomes were exhausted. The total number of wins and losses across all pairwise comparisons were aggregated to form the WR:

$$Win\;Ratio=\frac{Total\;wins\;\left(Xen\right)}{Total\;wins\;\left(Trab\right)}$$

A WR >1 indicates a relative advantage for Xen, while a ratio <1 favors Trab. Comparisons that remained tied after all hierarchical levels were exhausted were classified as “neutral pairs.” These neutral pairs are excluded from the numerator and denominator of the WR calculation. Confidence intervals and *P* values were estimated to assess statistical significance. To address the statistical dependence inherent in comparing the same patient multiple times, we employed asymptotic U-statistic theory for variance estimation. This method ensures that *P* values and confidence intervals are calculated based on subject-level data, effectively clustering the variance by patient to prevent overestimation of treatment effects. Patients with missing follow-up data were treated as censored at their last known visit (Finkelstein–Schoenfeld method), ensuring that their available data contributed to the pairwise comparisons up until the point of censoring.

#### *Sensitivity Analyses of Outcome Hierarchy*

Because the WR is dependent on the prespecified outcome hierarchy,^12^ we performed sensitivity analyses to test the robustness of the estimates. Two approaches were used.

1. Clinically defined permutations: we examined three alternative, plausible orderings of the hierarchy:
  o prioritizing vision-threatening complications above surgical reintervention (placing secondary glaucoma surgery last);
  o elevating bleb needling higher in the hierarchy;
  o restricting the analysis to a reduced composite of five outcomes.
2. Randomized permutations: To explore the impact of more arbitrary reweightings, we randomly permuted the order of outcomes 3–8 across 100 iterations.

## Results

### Cohort Characteristics

The baseline demographic and ophthalmic characteristics of the overall and matched cohorts are shown in Table 1. Among 1809 eyes in the Fight Glaucoma Blindness registry that underwent either Xen or Trab, 1257 eyes had at least 12 months of follow-up and were included. Propensity score matching yielded two balanced cohorts comprised of 212 eyes in the Xen and 212 eyes in the Trab groups. After matching, there were no significant differences in age, baseline IOP, medications, BCVA, central corneal thickness or VF MD between the Xen and Trab groups. Minor imbalances were observed in the diagnostic subtype; specifically, in the Xen group, there was a significantly lower proportion of eyes with Normal Tension Glaucoma and a higher proportion of eyes with Ocular Hypertension.

**Table 1:** Baseline Demographic Characteristics of Subjects and Eyes That Had Undergone Xen or Trab with 12 months of Follow-up in the Registry

Category	Level	Xen	Trab	*P* Value
Eyes		212	212	NA
IOP		22 (8.3)	22.3 (9.4)	0.7342
Medications		2.9 (1.2)	3 (1.2)	0.7905
VF MD		–9.9 (7.9)	–9.9 (7.7)	0.9415
BCVA (logMAR)		75.4 (15.2)	76.7 (11.6)	0.3316
CCT		532.3 (40.9)	533.6 (36.3)	0.7312
Diagnosis	Angle closure	13 (6.1%)	20 (9.4%)	0.277
	Normal tension glaucoma	15 (7.1%)	37 (17.5%)	0.002
	Ocular hypertension	15 (7.1%)	3 (1.4%)	0.006
	Open-angle glaucoma suspect	11 (5.2%)	2 (0.9%)	0.021
	Primary open-angle glaucoma	128 (60.4%)	113 (53.3%)	0.170
	Secondary open-angle glaucoma	30 (14.2%)	37 (17.5%)	0.425
Ethnicity	White/Caucasian	143 (67.5%)	113 (53.3%)	0.004
	Asian	20 (9.4%)	36 (17%)	0.031
	Other/unable to specify	49 (23.1%)	63 (29.7%)	0.152
Male gender		46.60%	60.40%	0.0013
Age		71.4 (11.4)	71.1 (10.8)	0.7404

BCVA = best-corrected visual acuity; CCT = central corneal thickness; IOP = intraocular pressure; logMAR = logarithm of the minimum angle of resolution; VF MD = visual field mean deviation.

### Efficacy Outcomes

Although the primary outcome of this study is assessing WRs, we first present standard efficacy outcome data to help contextualize these findings, which have been reported in part previously (Table 2).^13^ At 12 months eyes that underwent trabeculectomy had significantly lower IOP (11.7 ± 6.3 mmHg) than Xen (15.8 ± 8.3 mmHg; *P* < 0.001) and demonstrated greater absolute (–10.6 ± 10.1 mmHg vs. –6.2 ± 10.2 mmHg; *P* < 0.001) and percentage IOP reduction (–48.6% vs. –30.5%; *P* < 0.001). The proportion of eyes achieving complete success was higher for the Trab (58.5%) than for Xen (36.8%; *P* < 0.001), with a consistent difference across higher IOP thresholds. Qualified success (with medication) similarly favored trabeculectomy at ≤15 mmHg (62.7% vs. 47.6%; *P* = 0.002) and ≤18 mmHg (67% vs. 56.1%; *P* = 0.028).

**Table 2:** Efficacy Outcomes of Eyes That Had Undergone Xen or Trab with 12 months of Follow-Up in the Registry

Variable	Xen	Trab	*P* Value
Baseline IOP	22 (8.3)	22.3 (9.4)	0.734
Final IOP	15.8 (8.3)	11.7 (6.3)	<0.001
IOP change	–6.2 (10.2)	–10.6 (10.1)	<0.001
IOP % change	–30.5 (–50.4, –5.7)	–48.6 (–65.1, –21.9)	<0.001
Baseline meds	2.9 (1.2)	3 (1.2)	0.79
Final meds	1.3 (0.9)	1.1 (0.7)	0.004
Meds change	–1.6 (1.3)	–1.9 (1.2)	0.048
Qualified success 15 mmHg	101 (47.6%)	133 (62.7%)	0.002
Qualified success 18 mmHg	119 (56.1%)	142 (67%)	0.028
Qualified success 21 mmHg	125 (59%)	144 (67.9%)	0.07
Complete success 15	78 (36.8%)	124 (58.5%)	<0.001
Complete success 18	92 (43.4%)	131 (61.8%)	<0.001
Complete success 21	95 (44.8%)	133 (62.7%)	<0.001

IOP = intraocular pressure; Meds = medications; Trab = trabeculectomy; Xen = Xen gel stent implant.

Change in IOP and medications at 12 months from baseline are shown. Proportions of qualified and success at three upper IOP thresholds are also shown.

### Win Ratio

A total of 424 eyes were initially available for analysis. This allowed for 44 944 pairwise comparisons between groups. Across all hierarchical outcomes, trabeculectomy achieved more wins overall (18 118 vs. 15 074 for Xen) with 11 752 (26.1%) ties. This yielded an overall WR of 0.83 (95% confidence interval 0.63–1.10; *P*=0.19), indicating a numerical but nonsignificant advantage for trabeculectomy. At the highest-priority outcome (loss of light-perception vision), no events occurred. The next-ranked end point, secondary glaucoma surgery, favored trabeculectomy (4422 vs. 5772 wins in Xen vs. Trab, respectively). Among other early postoperative complications.

• Hypotony with ≥10-letter VA loss favored Xen (4806 vs. 4086 wins in Xen vs. Trab).
• Hypotony maculopathy (407 vs. 208 wins), choroidal effusion (344 vs. 337 wins), and hypotony alone (3646 vs. 3080 wins) favored Xen.
• Hyphaema with VA loss favored Trab (177 vs. 322 wins)
• Bleb needling favored Trab (1272 vs. 4314 wins in Xen vs. Trab).

The cumulative pairwise distribution of wins, ties, and losses across all outcomes, along with the observed incidence of each event is summarized in Table 3. Figure 2 shows the percentages of wins, losses, and ties at each level of the hierarchy, with the win difference at each level also displayed. The forest plots of WRs for each level and the overall estimate are shown in Figure 3.

**Table 3:** Pairwise Win–Loss Counts for Xen versus Trab across the Hierarchical Outcome Framework, alongside the Observed Incidence of Adverse Events in Each Group

Outcome	Number of Winners for Xen	Number of Losers for Xen	Incidence of Adverse Events	
			Xen (n = 212)	Trab (n = 212)
Loss of light perception vision (OC1)	0	0	0 (0.0%)	0 (0.0%)
Secondary glaucoma surgery (OC2)	4422	5772	39 (18.4%)	27 (12.7%)
Hypotony + VA loss ≥10 letters (0–6mo, OC3)	4806	4086	32 (15.1%)	34 (16.0%)
Hypotony maculopathy (0–6mo, OC4)	407	208	3 (1.4%)	6 (2.8%)
Choroidal effusions (0–6mo, OC5)	344	337	6 (2.8%)	12 (5.7%)
Hypotony alone (0–6mo, OC6)	3646	3080	63 (29.7%)	63 (29.7%)
Hyphaema + VA loss ≥10 letters (0–6mo, OC7)	177	322	5 (2.4%)	6 (2.8%)
Bleb needling (0–6mo, OC8)	1272	4313	61 (28.8%)	28 (13.2%)
	15 074	18 118	209 (98.6%)	176 (83.0%)

Trab = trabeculectomy; VA = visual acuity; Xen = Xen gel stent implant.

For each outcome, “winners for Xen” indicate pairwise comparisons in which Xen eyes had a more favorable result, while “losers for Xen” indicate comparisons in which trabeculectomy eyes were favored.

**Figure 2:**
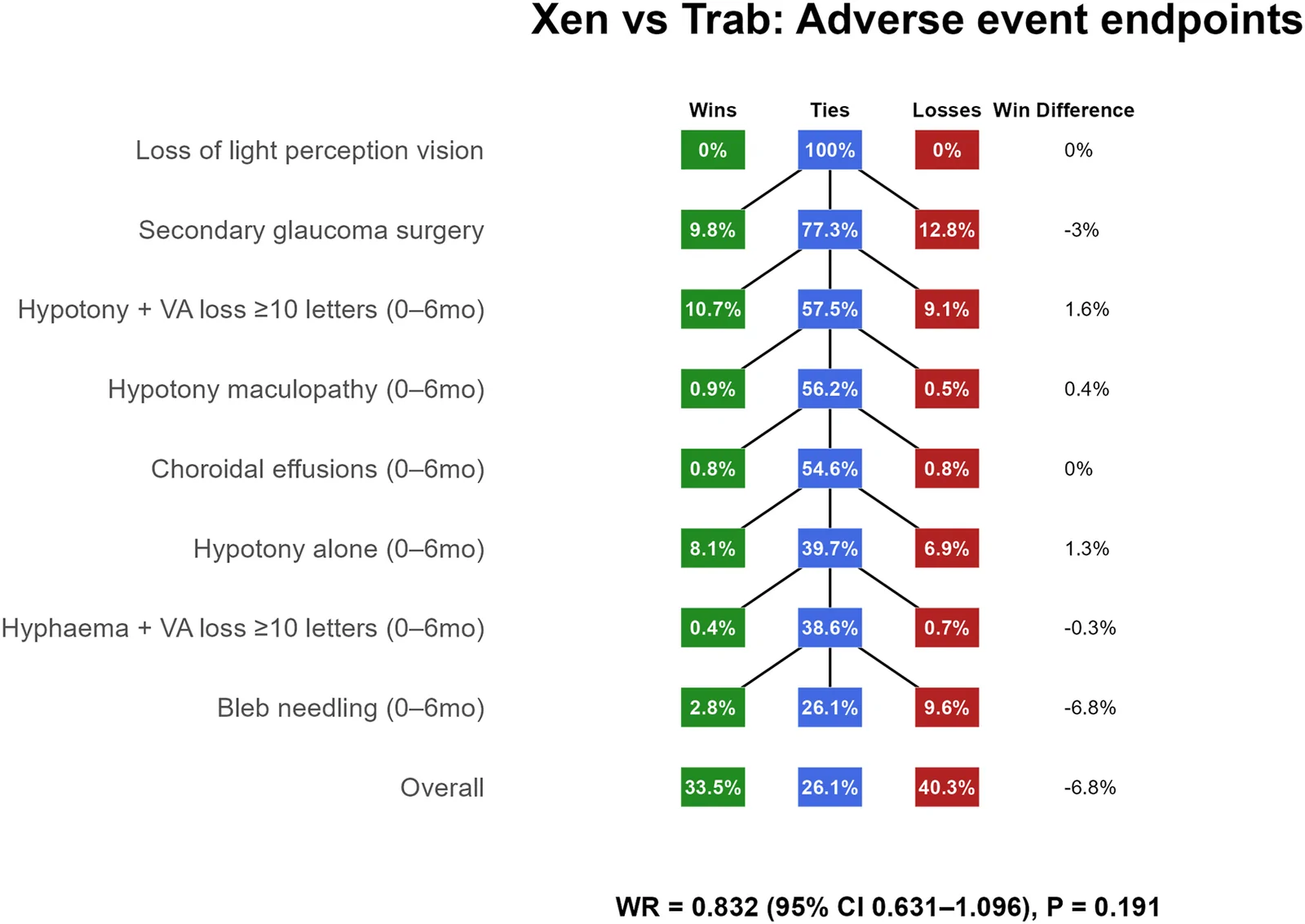
Hierarchical Win ratio analysis of adverse event endpoints comparing Xen versus Trab in the Fight Glaucoma Blindness registry. At each level, patient pairs are first evaluated for loss of light perception vision, then sequentially for secondary glaucoma surgery, adverse events (hypotony, choroidal effusions, hyphema), and finally the need for bleb needling. Each row displays the proportion of wins (green), ties (blue), and losses (red) for the treatment arm (Xen) in reference to the control arm (Trab) at that outcome level, with the far-right column showing the net win difference. A positive win difference therefore indicates Xen accumulated more wins than Trab at that level. The final row summarizes the overall win ratio, confidence interval, and *P* value. Trab = trabeculectomy; Xen = Xen gel stent implant.

**Figure 3:**
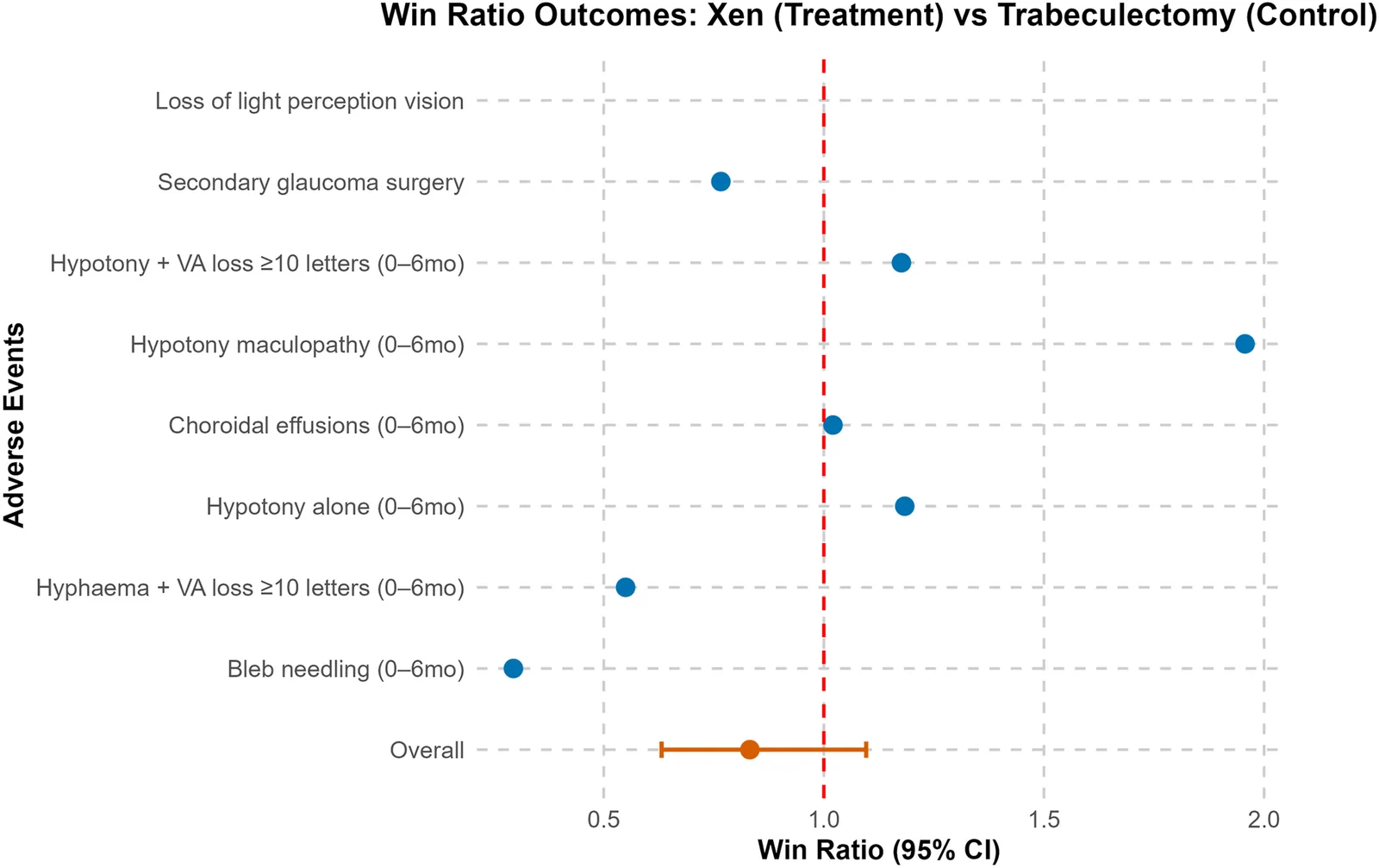
Forest plot of Win Ratios for Xen versus trabeculectomy across the hierarchical outcome framework. Each point represents the Win Ratio for an individual adverse event, with values >1 favoring Xen and <1 favoring trabeculectomy. The overall Win Ratio (orange; 0.83) is shown at the bottom, with error bars denoting 95% confidence intervals. The vertical dashed line at 1.0 represents the line of no difference. The overall Win Ratio of 0.83 (95% CI 0.63–1.10; *P* = 0.19) for Xen indicates a numerical but nonsignificant advantage for Trab compared to the treatment arm. CI = confidence interval; Trab = trabeculectomy; Xen = Xen gel stent implant.

### Sensitivity Analyses

To test the sensitivity of the WR estimate to outcome ordering, we explored both manually defined permutations (Fig 4) and randomly sampled permutations (Fig 5). In Figure 4, we examined three clinically plausible rearrangements.

i. prioritizing vision-threatening complications over surgical re-intervention (secondary glaucoma surgery placed last);
ii. elevating bleb needling in the hierarchy; and
iii. focusing on a reduced composite of five outcomes.

**Figure 4:**
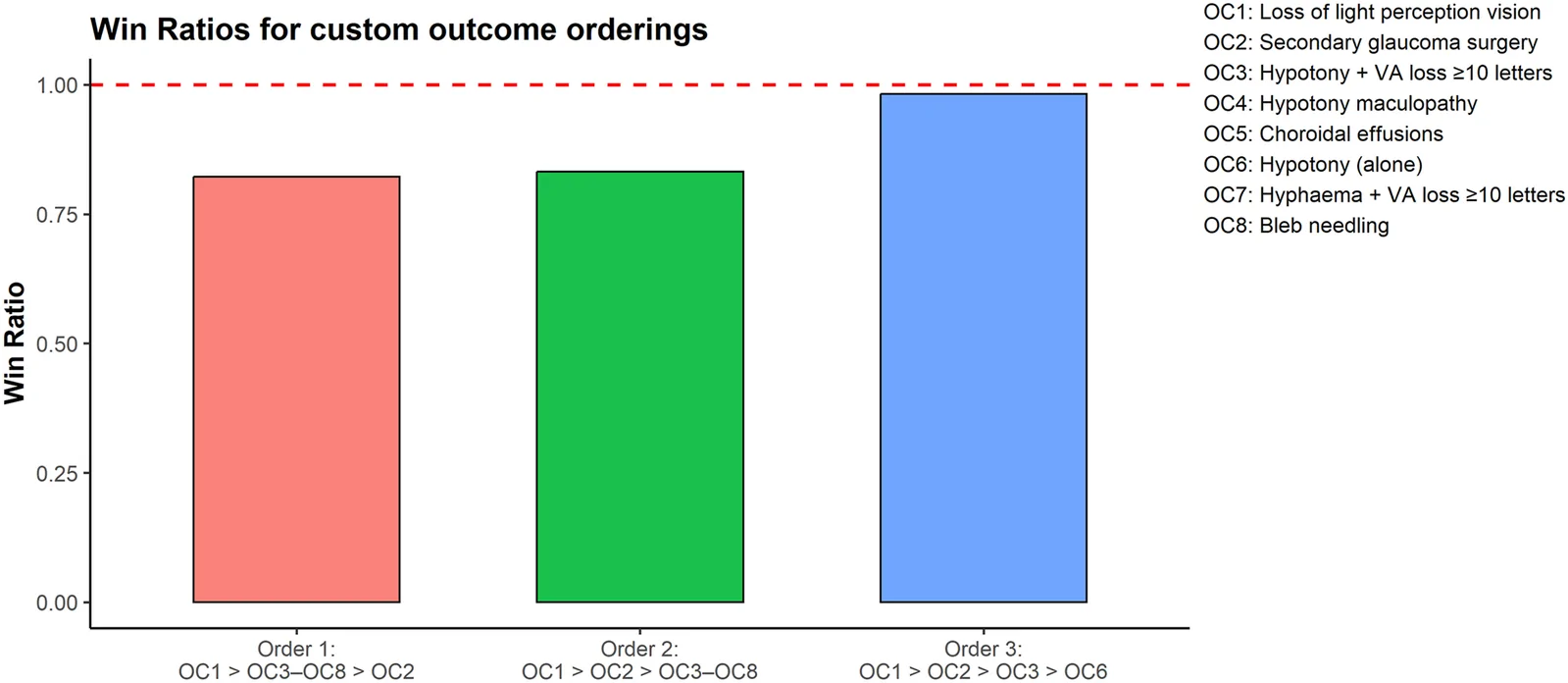
Win Ratios for Xen versus trabeculectomy under three alternative outcome hierarchies. Order 1 prioritized vision-threatening complications ahead of secondary surgery; order 2 elevated secondary surgery earlier in the hierarchy; and order 3 restricted the analysis to a reduced composite of five outcomes. In all scenarios, Win Ratios ranged between 0.82 to 0.98, which indicates the Win Ratio from each hierarchy favoured trabeculectomy over Xen. Trab = trabeculectomy; Xen = Xen gel stent implant.

**Figure 5:**
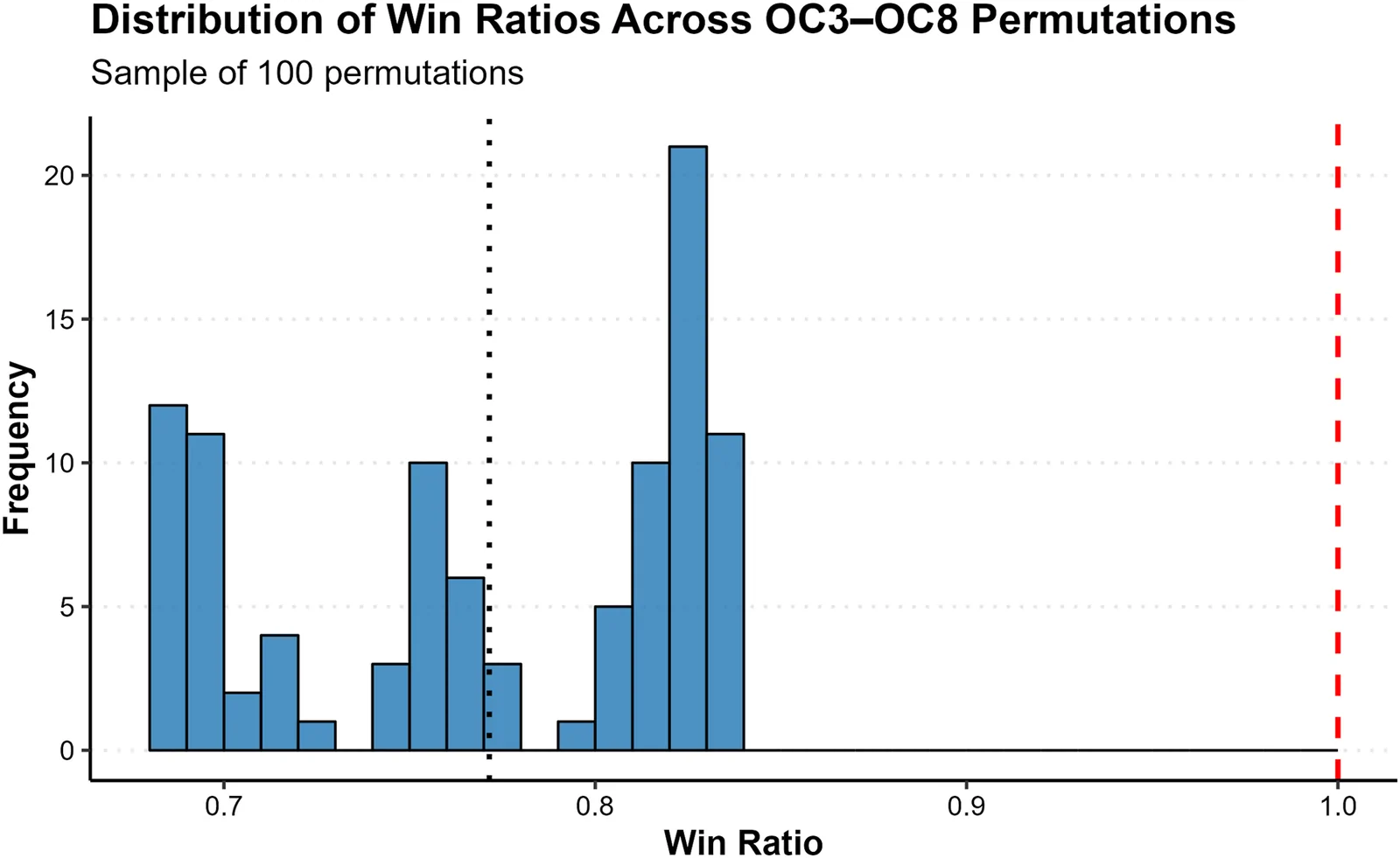
Distribution of Win Ratios from 100 random permutations of outcomes OC3–OC8 (hypotony with vision loss, hypotony maculopathy, choroidal effusions, numerical hypotony, hyphaema and vision loss, and bleb needling). Each bar represents the frequency of Win Ratios observed under different outcome orderings. The dotted vertical line marks the median Win Ratio (0.77), while the dashed red line at 1.0 represents the line of no difference. Across all permutations, Win Ratios consistently clustered below 1.0, indicating stable inferiority of Xen compared with trabeculectomy regardless of outcome ordering. Xen = Xen gel stent implant.

The three orderings resulted in WRs between 0.82 and 0.98, which still favored Trab. The removal of bleb needling notably moved the direction of the ratio in favor of Xen. This nevertheless highlights the robustness of the treatment effect across reasonable alternative hierarchies.

In Figure 5, we randomly permuted the order of outcomes 3 to 9 across 100 iterations. The resulting histogram of WRs demonstrated a clustering around 0.77, with no permutations favoring Xen. This permutation-based analysis affirms that even if the specific hierarchy of adverse events is uncertain or debated, the conclusion of Xen having an inferior safety profile is robust.

## Discussion

In this study, we applied the WR method to compare the safety profile of Xen and Trab using a hierarchical composite framework that weighted adverse events by clinical importance. This approach ensures that more serious complications are given greater weight in determining the overall outcome. This study provides the first proof-of-concept for the WR approach in ophthalmology, illustrating its potential capacity to integrate multiple outcomes of unequal severity within a single, interpretable metric.

Traditional analyses in glaucoma surgery—whether based on time-to-first-event or single-endpoint comparisons—treat disparate events as equivalent and often fail to capture the relative clinical gravity of outcomes. For example, the loss of light perception clearly outweighs the need for a secondary procedure, yet both would contribute equally to a simple composite endpoint. Moreover, the timing of adverse events can meaningfully influence prognosis but is often ignored by conventional methods. The WR allows analysis of composite endpoints prioritizing clinically critical outcomes and permitting time-to-event evaluation within a unified framework.^6^ Two approaches can be used to form the patient pairs for comparison.^6^ The first is a matched pair approach, where each treated patient is compared with a specific control, giving only 1 comparison for each subject. Patients in the treatment and control groups (or new and standard treatment groups) are paired according to baseline risk, and each pair is compared for occurrences of events using the clinical hierarchy of outcomes.^6^ The second method of pairing is an unmatched (or all-pairs) approach, where each treated patient is compared with all controls, maximizing the number of comparisons. This method produces N_t_ x N_c_ paired comparisons followed by classifying all possible pairs using the hierarchy,^6^ and was used in our study. Within each pair, outcomes are then evaluated in descending order of clinical importance until 1 patient is judged to have fared better. A better outcome for the new treatment is counted as a “win,” while a better outcome for the control is counted as a “loss”; if no difference is observed, the pair is considered a tie. Summing across all comparisons yields the total number of wins (N_W_), losses (N_L_), and ties, with the WR calculated as N_W_/N_L_.^6^

### Composite versus Hierarchical Outcomes

Since its release, the WR has influenced the methodology and interpretation of cardiovascular trials by providing a more clinically meaningful assessment of treatment outcomes. It has consequently become more widely incorporated into trial design. For instance, in a systematic review of cardiovascular randomized trials published between January 2022 and July 2024, 36 eligible randomized clinical trials were identified that applied the WR, most commonly in heart failure and ischemic heart disease.^7^

Like many specialities, ophthalmology often involves interventions with risks and outcomes of differing importance, with most trials reporting a range of efficacy and safety outcomes either separately or as simple composites.^1^, ^2^, ^3^ The present analysis demonstrates that this approach may be adaptable to ophthalmology, where treatments frequently involve trade-offs between efficacy and safety and where event rates are low and heterogeneous. In landmark glaucoma studies, such as the tube versus trabeculectomy trial,^9^ endpoints were defined as binary “success” or “failure” based on thresholds for IOP reduction, reoperation, or vision loss. Such definitions simplify analyses but obscure the clinical nuance that a mild pressure elevation is less consequential than irreversible blindness. A hierarchical WR approach could reorder these outcomes by severity, yielding a more intuitive reflection of treatment benefit or harm, and thus assist application into clinical practice.

Our results parallel this reasoning: despite higher early intervention rates in Xen, catastrophic vision-threatening events were rare in both procedures. The WR synthesized these observations into a single effect estimate (WR = 0.83) that appropriately reflected the dominance of less severe but more frequent complications in determining the overall trend.^6^ For GFS, such a hierarchy might reasonably prioritize loss of light perception over the need for secondary glaucoma surgery, and the latter over transient hypotony. Applying this approach, our analysis demonstrated that trabeculectomy overall accumulated more wins compared with Xen, although this difference was not significant. Furthermore, displaying the WR for each component of the hierarchy enables readers to identify which component outcomes drive the overall effect, as illustrated in Figure 2.

The WR can accommodate not only safety outcomes but also efficacy outcomes, allowing both to be integrated within a single hierarchical framework. For example, in GFS efficacy, can be defined by IOP reduction using different upper thresholds as recommended by the World Glaucoma Association, alongside safety outcomes including vision loss, hypotony, or the need for reoperation. This structure enables efficacy and safety to be considered together, as opposed to traditional analyses that often report these outcomes separately. Although we have used an exemplar of glaucoma, the WR as an analytic tool is likely to have utility across most other areas of ophthalmology. For instance, in neovascular age-related macular degeneration trials, visual acuity gains could be analyzed alongside key safety outcomes such as uveitis or endophthalmitis. The ANCHOR study compared ranibizumab with photodynamic therapy in neovascular age-related macular degeneration.^14^ The primary endpoint was the proportion of eyes losing fewer than 15 letters at 1 year, with secondary endpoints including visual acuity gain/loss and rates of adverse events such as endophthalmitis or uveitis reported separately.^14^ Applying the WR would enable a hierarchical, integrated assessment of these endpoints, and could, for instance, prioritize vision-threatening adverse events before visual preservation for a more clinically intuitive comparison of overall treatment benefit.

### Time Horizon and the Hierarchy

A potential criticism of a hierarchical comparison is that it reports a treatment difference over a fixed period of observation. While that criticism applies to any endpoint, whether a single outcome, a conventional composite or a hazard ratio, it has particular force in a WR because of the way the hierarchy resolves. A lower level is only consulted when every level above it is tied, so which component decides a given pair depends on how many pairs reach that level, and that distribution is a function of follow-up duration. As observation lengthens, late and severe events do not simply add to the result; they pre-empt the efficacy comparisons beneath them, and the implicit weighting of the components changes even though the prespecified hierarchy does not. In glaucoma surgery, the components have systematically different time courses, so this drift may well be substantial rather than marginal. Hypotony occurs early and usually resolves within months, loss of IOP control accrues progressively over years, and irreversible loss of vision is uncommon and late. The balance between safety and efficacy within the composite is therefore a property of the horizon chosen as much as of the procedures being compared.

The Advanced Glaucoma Intervention Study illustrates this. Eyes uncontrolled on medical therapy were randomized either to argon laser trabeculoplasty followed by Trab or to Trab followed by argon laser trabeculoplasty, and were followed for 7 years.^15^ The reduction in IOP was greater and failure of the initial intervention less common in eyes randomized to Trab first, yet in 1 subgroup the visual field outcome favored the laser first sequence through the first year and the Trab first sequence for every year thereafter. Had a hierarchical analysis been applied, the level on which most pairs were decided would have moved upward as the study matured, and on the visual field component the direction of the result would have depended on when the comparison was made. We would argue that the hierarchy and the observation period are a single prespecified decision rather than two, and that the level at which each pair is resolved should always be reported so that readers can see which components carried the result and can reweight them as technique and technology change.

### Limitations

Several recognized limitations of the WR warrant consideration. First, the method depends on the definition of a clinically meaningful hierarchy and requires expert consensus, which introduces a degree of subjectivity into the analysis. This includes agreement on the number and the structure of hierarchy levels and standardization of heterogeneous efficacy endpoints,^16^^,^^17^ the order of which may lead to varying results. Patient perspectives on the hierarchy of surgical outcomes are also incompletely understood and need consideration alongside surgeon expert consensus.^18^ Our analyses investigating the effect of changing the hierarchy, however, showed only a modest change in the headline WR, and the overall direction of effect consistently favored trabeculectomy. This underscores the potential strength of the metric, as the overall conclusion remained stable despite differences in how outcomes are prioritized. This stability applies to reordering within a fixed 12-month window and does not extend to the choice of observation period itself. A further potential limitation is that the analysis is dependent on the quality of the underlying registry data, specifically that adverse events may have been under-reported in our dataset. While such under-reporting would be expected to affect both arms similarly, the importance of clean, high-quality datasets in observational data^19^ is essential for accurately interpreting the WR. Finally, this analysis focused primarily on short-term safety outcomes, whereas longer-term endpoints such as late bleb failure or endophthalmitis were not captured. As set out above, the level at which pairs resolve depends on the duration of observation, so our result should be read as a comparison at 12 months rather than a general ordering of the two procedures. Given that the primary goal of GFS is to provide long-term IOP control, these delayed safety endpoints are of particular importance and should be integrated in future studies.^7^

Regarding the characteristics of our cohort, an important limitation is the difference in diagnostic subtypes, ethnicity, and sex between both groups. For instance, there was a significantly higher proportion of eyes with normal tension glaucoma in the Trab group. This imbalance likely reflects surgeon-driven selection bias in a real-world clinical setting, where the choice of procedure is often tailored to the specific glaucoma phenotype. While propensity score matching was used to balance key baseline variables such as preoperative IOP, number of medications, and visual field loss, we did not match by other characteristics such as diagnostic subtypes to maintain a robust sample size for the WR analysis. However, these baselines imbalances should be considered when interpreting the comparative outcomes. Furthermore, the surgical techniques utilized in this cohort were not standardized and reflect the clinical practices of the study period. Surgical success in Xen has been shown to be influenced by the technique of implantation—open vs. closed conjunctival, ab interno vs. ab externo,^20^^,^^21^ and dose of adjunctive agents like mitomycin-C.^17^^,^^22^ As surgical techniques for glaucoma drainage procedures continue to refine, the comparative risk/benefit profiles and outcomes for one procedure over another will likely shift.

Overall, we demonstrate the utility of the WR as a clinically meaningful addition to conventional analyses, integrating multiple outcomes within a hierarchy that respects clinical priorities. It is offered as a further means of examining trial data rather than a replacement for current methods of evaluating glaucoma surgery, and its incremental value in this setting remains to be established. While the current hierarchy focused primarily on safety and surgical failure, the incorporation of long-term efficacy—such as sustained IOP reduction and medication-free success—is an essential next step in developing WRs for use in glaucoma and ophthalmology more generally. Broader adoption of the metric will likely depend on consensus-driven hierarchies, ideally shaped by expert panels which incorporate both clinician and patient perspectives. Prospective randomized trials could then subsequently incorporate the WR methodology in their design to better assess its utility in ophthalmology.
